# The challenge of translating ischemic conditioning from animal models to humans: the role of comorbidities

**DOI:** 10.1242/dmm.016741

**Published:** 2014-12

**Authors:** Kieran McCafferty, Suzanne Forbes, Christoph Thiemermann, Muhammad M. Yaqoob

**Affiliations:** Translational Medicine and Therapeutics, William Harvey Research Institute, Queen Mary University London, London, EC1M 6BQ, UK.

**Keywords:** Comorbidities, Ischemic postconditioning, Ischemic preconditioning, Remote ischemic preconditioning

## Abstract

Following a period of ischemia (local restriction of blood supply to a tissue), the restoration of blood supply to the affected area causes significant tissue damage. This is known as ischemia-reperfusion injury (IRI) and is a central pathological mechanism contributing to many common disease states. The medical complications caused by IRI in individuals with cerebrovascular or heart disease are a leading cause of death in developed countries. IRI is also of crucial importance in fields as diverse as solid organ transplantation, acute kidney injury and following major surgery, where post-operative organ dysfunction is a major cause of morbidity and mortality. Given its clinical impact, novel interventions are urgently needed to minimize the effects of IRI, not least to save lives but also to reduce healthcare costs. In this Review, we examine the experimental technique of ischemic conditioning, which entails exposing organs or tissues to brief sub-lethal episodes of ischemia and reperfusion, before, during or after a lethal ischemic insult. This approach has been found to confer profound tissue protection against IRI. We discuss the translation of ischemic conditioning strategies from bench to bedside, and highlight where transition into human clinical studies has been less successful than in animal models, reviewing potential reasons for this. We explore the challenges that preclude more extensive clinical translation of these strategies and emphasize the role that underlying comorbidities have in altering the efficacy of these strategies in improving patient outcomes.

## Introduction

Ischemia-reperfusion injury (IRI) refers to the damage caused when blood supply is restored to a tissue after a period of restricted blood flow (ischemia) (see Box 1 for a glossary of terms). It is a pathological mechanism that contributes to many common clinical conditions. Although the heart is the organ in which IRI has been mostly researched, the relevance of IRI is by no means limited to ischemic heart disease, but is pertinent to any disease state in which blood supply to an organ or tissue is compromised and then restored, including cerebrovascular disease, acute kidney injury (AKI; see Box 1), solid organ transplantation and post-surgery organ dysfunction.

**Box 1. Glossary**

Acute kidney injury (AKI): a rapid and significant deterioration of kidney function.
Autacoids: locally produced substances that act on other cells in the same region.
Calcitonin gene-related peptide (CGRP): a neuropeptide widely distributed in the nervous and cardiovascular systems with a role in cardiac hypertrophy.
Caspase-3: a member of the cysteine-aspartic acid protease family involved in the execution phase of apoptosis.
Chronic kidney disease (CKD): progressive and long-term loss of kidney function.
Coronary angioplasty: a procedure that is used to widen blocked or narrowed coronary arteries.
Cytoprotective: the ability of an agent (pharmacological or biological) to protect cells from otherwise harmful stimuli.
Extracellular signal-regulated kinase (ERK)1/2: members of the mitogen-activated protein-kinase superfamily; intracellular signalling molecules involved in mediating cell proliferation and apoptosis.
Glycogen synthase kinase (GSK)-3β: an isoform of GSK-3, a serine-threonine kinase enzyme involved in many intracellular signalling pathways.
Heat shock protein (HSP)-70: one of a group of proteins induced by heat shock.
Ischemia: restriction of blood supply to a tissue.
Ischemic conditioning: the process of exposing a particular organ or tissue to brief periods of sub-lethal ischemia, followed by reperfusion.
Ischemic post-conditioning (iPOST): refers to ischemic conditioning after an episode of ischemia.
Ischemic preconditioning (IPC): refers to ischemic conditioning before an episode of ischemia.
JAK-STAT pathway: a key signalling pathway for many cytokines and growth factors that stimulates cell proliferation, differentiation, migration and apoptosis. JAK stands for Janus kinase and STAT for signal transducer and activator of transcription.
K_ATP_: adenosine triphosphate (ATP)-dependent potassium channel found in the plasma or in subcellular (mitochondrial or sarcolemmal) membranes.
Mevalonate pathway: also known as the HMG-CoA reductase pathway, an important metabolic pathway involved in synthesizing cholesterol and other sterol/non-sterol isoprenoids.
Mitochondrial permeability transition pore (MPTP): a protein pore in the inner membrane of the mitochondria formed under certain conditions; it allows increased permeability, with subsequent swelling and cell death.
Mitogen-activated protein kinase phosphatase-1 (MKP-1): a phosphatase involved in the negative regulation of cellular proliferation.
Myocardial matrix metalloproteinase (MMP)-2: a proteolytic enzyme leading to extracellular-matrix degradation.
NSTEMI: non-ST segment elevation myocardial infarction; it refers to a particular electrocardiogram pattern seen in patients with an acute myocardial infarction.
Peroxynitrite-induced nitrosative stress: enhanced peroxynitrite formation that results in increased cellular damage through its oxidant properties.
Phosphatase and tensin homologue (PTEN): a phosphatase involved in cell cycle regulation.
Phosphatidylinositol-3-kinase–Akt (PI3K-Akt): a pathway involved in cell proliferation and survival.
Primary coronary intervention (PCI): a coronary angioplasty to open the section of narrowed coronary artery that is causing ischemia.
Protein kinase C: family of enzymes involved in many intracellular signal transduction mechanisms.
Remote ischemic per-conditioning: ischemic conditioning in an organ remote to the site of an episode of ischemia, during the target organ ischemia.
Remote ischemic post-conditioning: refers to ischemic conditioning in an organ remote to the site of an episode of ischemia, and after the ischemic insult.
Remote ischemic preconditioning (RIPC): refers to ischemic conditioning in an organ remote to the site of an episode of ischemia, and before the ischemic insult.
Reperfusion injury salvage kinase (RISK) pathway: a pathway activated at the time of reperfusion and involved in protection from reperfusion injury.
Streptozotocin: a chemical that is toxic to the pancreas and used in generating animal models of type 1 diabetes.
Survivor activating factor enhancement (SAFE) pathway: a pathway activated at the time of reperfusion and involved in protection from reperfusion injury.
Tumour necrosis factor (TNF)α: a cytokine involved in systemic inflammation.

A quarter of a century ago, a seminal paper reported that brief episodes of non-lethal ischemia applied to the circumflex artery in a dog model of acute myocardial infarction, followed by its reperfusion, could render the myocardium profoundly resistant to ensuing prolonged ischemia, reducing subsequent infarct size by 75% (Murry et al., 1986). The term preconditioning with ischemia, or ischemic preconditioning (IPC; see Box 1), was used to describe this phenomenon. IPC was subsequently shown, by *in vivo* studies, to be effective in other species, including pigs (Schott et al., 1990), rabbits (Thornton et al., 1990), rats (Yellon et al., 1992), sheep (Burns et al., 1996) and mice (Sumeray and Yellon, 1998; Xi et al., 1998). Furthermore, the beneficial effect of IPC was not myocardium-specific, with studies demonstrating that other tissues, including the lung (Li et al., 1999), liver (Hardy et al., 1996) and kidneys (Cochrane et al., 1999), gained ischemia tolerance from IPC.

Seven years after this seminal paper, Przyklenk et al. reported that IPC, used in an *in vivo* dog model of myocardial ischemia, could reduce infarct size by 70% and proposed that a signal from IPC could also trigger tissue protection in distant vascular beds (Przyklenk et al., 1993). The demonstration of this distal protective effect led them to coin the term remote ischemic preconditioning (RIPC; see Box 1). As with IPC, RIPC was subsequently found to have ubiquitous tissue-protective properties in *in vivo* experiments, across organs and species. For example, brief episodes of ischemia and reperfusion of the leg could be used to precondition the kidney in a rat model of renal IRI (Wever et al., 2013), the liver in a rabbit model of hepatic IRI (Kanoria et al., 2006), and the brain in a pig model of cardiopulmonary bypass (Jensen et al., 2011).

A decade later, Zhao et al. demonstrated that intervening at the point of reperfusion with additional brief episodes of ischemia and reperfusion could confer tissue protection. They reported a 44% reduction in infarct size in a canine model of myocardial ischemia and reperfusion (Zhao et al., 2003) and termed this ischemic post-conditioning (iPOST; see Box 1). As with the other ischemic conditioning strategies initially described in the heart, iPOST has since been reproduced in various organs across various species (Jiang et al., 2010; Zhao et al., 2006).

**Fig. 1.**
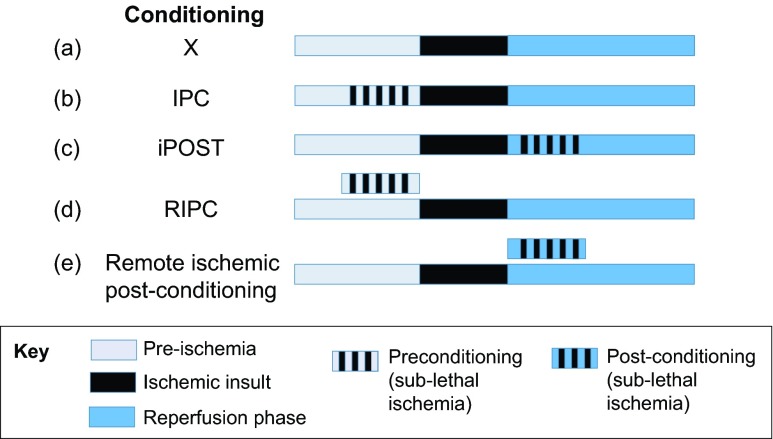
**Schematic diagram of IPC, iPOST and RIPC protocols.** Schematic representation of the differing protocols of ischemic conditioning: light blue represents pre-ischemia; black the ischemic insult; black lines represent the application of sub-lethal ischemia; dark blue the reperfusion phase. (a) Ischemia-reperfusion with no ischemic conditioning. (b) Ischemic preconditioning (IPC), with sub-lethal ischemia applied before the insult (black lines). (c) Ischemic post-conditioning (iPOST), with sub-lethal ischemia applied after the insult (black lines). (d) Remote ischemic preconditioning (RIPC), where the sub-lethal ischemia is applied distal to and prior to the area and time of ischemia. (e) Remote ischemic post-conditioning, where the sub-lethal ischemia is applied distal to and subsequent to the area and time of ischemia.

Ischemic conditioning protocols have now been extended to include remote ischemic per-conditioning (Szijártó et al., 2012) and remote ischemic post-conditioning (Vinten-Johansen and Shi, 2013), whereby the applied sub-lethal episodes of ischemia and reperfusion in the distant organ starts after the initial ischemic episode or after reperfusion, respectively (see Box 1 and Fig. 1). Different ischemic conditioning protocols have also been combined to provide additional tissue protection (Sato et al., 2007; Song et al., 2012; Xin et al., 2010).

Among the many experimental strategies that have been investigated to reduce IRI, IPC, RIPC and iPOST seem to be ideal candidates to take forward into human clinical trials: they are safe, simple to perform and reproducible. In animal models, they also produce some of the most potent cytoprotective (see Box 1) interventions in the field of ischemia-reperfusion biology, attenuating tissue injury by up to 80% (Murry et al., 1986). As discussed and illustrated in Box 2, considerable progress has been made in recent years to elucidate the mechanisms by which the brief episodes of ischemia and reperfusion associated with IPC, RIPC and iPOST lead to tissue protection.

Since 1993, when the first study of IPC in humans was published (Yellon et al., 1993), there have been over 150 clinical trials of IPC, RIPC and iPOST techniques. The main initial limitation to the clinical use of IPC was the need to directly access the blood supply of the organ at risk. RIPC overcomes this, allowing preconditioning of internal organs and tissues using intermittent inflations of a blood-pressure cuff around a limb, rendering it transiently ischemic. A further limitation was that both IPC and RIPC had to be applied in advance of the lethal ischemic episode, making them applicable only to controlled elective cases. For example, IPC could not be a routine treatment of acute myocardial infarction – the event is not planned and there is no immediate access to the coronary circulation. iPOST overcomes this by enabling intervention at the time of reperfusion. The huge benefit, therefore, of iPOST and RIPC is that they can offer similar protection as IPC but applied after or distal to the event.

Accordingly, RIPC and iPOST have been investigated as clinical strategies in a large number of clinical trials, primarily for cardiac protection in the context of coronary artery bypass grafting, valve replacements or percutaneous coronary intervention (PCI). However, they are also protective in many other different settings, including: liver and kidney transplantation (Selzner et al., 2012), major abdominal surgery (Ali et al., 2007), AKI (Er et al., 2012) and stroke (Hougaard et al., 2014). Despite this, these techniques have not become part of routine clinical practice, potentially for two reasons. First, many of the trials performed over the last two decades were small, single-centre studies, frequently with fewer than 100 participants and a short follow-up period. As such, they have lacked the power needed to investigate the hard end points of death or a major cardiovascular event. Recently, however, one group have shown that the beneficial effects of RIPC in the context of elective PCI remain significant even 6 years after the event (Davies et al., 2013). Second, the effectiveness of ischemic-conditioning strategies in humans seems to be less profound than reported in the animal literature, with some randomized clinical trials showing no significant benefit (Abdelnoor et al., 2014; Brevoord et al., 2012). A possible explanation for this is the effect of underlying comorbidities on the ability of tissues to respond to the beneficial effects of ischemic conditioning.

When extrapolating from animal models to humans it is vital to understand the differences between animal models and patients. Animals used for *in vivo* work are often juvenile, derived from inbred strains, of the same age and health, housed in the same environment with identical diets, and have no comorbidities. Compare this to individuals with cardiovascular disease who participate in clinical trials, who are typically older, with comorbidities including diabetes, hypertension and kidney disease, and are taking several medications. Could it be that this disparity is the key to understanding why ischemic conditioning strategies fail to translate from animals to humans?

In this Review, we explore the role that such comorbidities have in reducing the clinical effectiveness of ischemic conditioning strategies. We examine several major comorbidities and their potential negative impact, beginning with hypercholesterolemia. For each condition, we discuss the clinical problem, the results of studies looking at IPC, RIPC and iPOST in animal models in that setting, and the outcomes of any relevant human studies. We close by discussing the difficulties in translating IPC into a useful clinical strategy, and potential avenues for future study.

## Hypercholesterolemia

### The condition

Hypercholesterolemia (or hyperlipidemia) describes high levels of cholesterol (lipids) in the blood. The relationship between hypercholesterolemia and cardiovascular mortality is well established (Shekelle et al., 1981), with elevated low-density lipoprotein (LDL) cholesterol long identified as a primary risk factor for coronary artery disease (Eisenberg, 1998). Hypercholesterolemia is, despite public health efforts, still common and an important cardiovascular risk factor (Ford et al., 2010). Statin treatment to lower serum cholesterol is a key part of both primary and secondary prevention of cardiac events. Experimental hypercholesterolemia has been shown to render animal hearts more susceptible to ischemic insult (Kocić et al., 1998).

### Effects on preconditioning strategies

Hypercholesterolemia was the first comorbidity reported to alter responses to preconditioning, with hypercholesterolemic rabbits unable to respond to IPC in a model of pacing-induced preconditioning (Ferdinandy et al., 1998; Szilvassy et al., 1995). The first report of IPC in hypercholesterolemic rats found that isolated papillary muscle from rats fed a high-fat diet was more susceptible to the effects of ischemia and less protected by the effects of IPC compared with control rats (Kocić et al., 1999). Since then, several potential mechanisms have been proposed to explain the lack of a preconditioning effect.

Tang et al. found that the effects of IPC were lost in an *in vivo* rabbit model of hypercholesterolemia, owing to impaired upregulation of tetrahydrobiopterin (BH4), which is essential for inducible nitric oxide (NO) synthase (Tang et al., 2005). However, an interesting experiment looking at the effects of both IPC and iPOST on infarct size in rabbits found, contrary to the studies above, that the effects of IPC were preserved in hypercholesterolemic hearts, but lost in post-conditioned animals, using two different protocols for iPOST (Iliodromitis et al., 2006). Kupai et al. also noted the abrogation of the tissue-protective effects of iPOST following a myocardial infarction in hypercholesterolemic rats, and sought to explain this by examining the protective role of early peroxynitrite-induced nitrosative stress (see Box 1) and the lack of this mechanism in the hypercholesterolemic heart (Kupai et al., 2009). Others, however, have disagreed with these findings, suggesting that the effects of iPOST, as well as IPC, were preserved in isolated rabbit hearts through the activation of the adenosine A1 receptor and K_ATP_ channels (see Box 1) (Donato et al., 2007).

In light of the loss of NO bioavailability and increased formation of peroxynitrite in hypercholesterolemic models, the effects of myocardial matrix metalloproteinase (MMP)-2 (see Box 1) have also been examined. Isolated rat hearts from cholesterol-fed Wistar rats were used to show that the protective inhibition of MMP-2 that is generated by IPC is blocked in hypercholesterolemic animals, and that a reduction in infarct size can be produced using an MMP inhibitor in non-preconditioned hearts (Giricz et al., 2006). More recently, the effect of IPC on cardiac gene expression has been examined, specifically looking at genes involved in NO and free radical signalling and in the mevalonate pathway (see Box 1), through which statins exert their effect (Kocsis et al., 2010).

Other factors proposed in the loss of the cardioprotective benefit of IPC include heat shock protein (HSP)-70 (see Box 1), which is downregulated post-transcriptionally in hypercholesterolemia (Csont et al., 2002), and caspase-3, the activation of which is increased in hypercholesterolemic, ischemic rabbit myocardium and which has a central role in cell apoptosis (Wang et al., 2002).

### Studies in humans

There are no published studies in either animal models or humans that examine the effects of RIPC on the hypercholesterolemic heart. However, two clinical studies have investigated the effect of iPOST in the context of hypercholesterolemia. These examined the effects of repeated balloon inflations at the time of angioplasty in patients with coronary artery disease (Kyriakides et al., 2002; Ungi et al., 2005) and looked at electrocardiogram (ECG) changes related to ischemia during the procedure as the outcome. Both trials reported that the cardioprotective effect of IPC was lost in the higher-cholesterol patient groups.

## Diabetes

### The condition

Diabetes is a metabolic disease of insulin deficiency or insulin resistance, associated with both micro- and macrovascular complications. Microvascular complications include diabetic retinopathy, nephropathy and neuropathy, whereas macrovascular complications include cardiovascular disease, peripheral vascular disease and stroke. The prevalence of diabetes continues to rise, and it is estimated that up to 10% of the population in the United States are diabetic (CDC, 2014). Both type 1 and type 2 diabetes are potent risk factors for the development of cardiovascular disease (Kannel and McGee, 1979), and ischemic heart disease is the major source of morbidity and mortality in these patients, who are not only two to four times more likely to have a cardiac event than the general population (Franco et al., 2007; Sarwar et al., 2010) but also have a worse prognosis following this event (Abbott et al., 1988; Haffner et al., 1998).

### Effects on preconditioning strategies

Although some animal studies report that diabetes is not a barrier to IPC (Ravingerová et al., 2000; Tatsumi et al., 1998), the majority report that the diabetic heart (Tosaki et al., 1996; Kersten et al., 2000; Nieszner et al., 2002) and the acutely hyperglycemic heart (Kersten et al., 1998) are resistant to IPC. Several pathways are implicated in this lack of response, including impaired phosphorylation of PI3K-Akt, decreased generation of NO and eNOS, abnormal ERK1/2 activity, K_ATP_ dysfunction, and reduced release of calcitonin gene-related peptide (CGRP; see Box 2 and Box 1 for more on these factors) (Rana et al., 2014).

**Box 2. Schematic representation of the transduction pathways of IPC, RIPC and iPOST**

Here, we provide a brief overview of the mechanisms involved in ischemic conditioning events and direct readers to several excellent, recent reviews for further information (Hausenloy, 2013; Lecour, 2009; Ovize et al., 2010; Przyklenk and Whittaker, 2013; Sluijter et al., 2014; Vinten-Johansen and Shi, 2011).

**IPC and iPOST mechanism**

➢ Transient episodes of ischemia generate:
  - autacoids (including adenosine, opioids and bradykinin), which bind to cell-surface G-protein-coupled receptors (G-PCRs);
  - tumour necrosis factor-α (TNFα), which binds to TNF-receptors (TNF-Rs).
➢ Activation of G-PCRs leads to the induction of the reperfusion-injury salvage kinase (RISK) pathway, a pro-survival pathway.
➢ Activation of TNF-Rs leads to the induction the survivor activating factor enhancement (SAFE) pathway, a pro-survival pathway.
➢ RISK pathway:
  - accumulation of pro-survival, anti-apoptotic protein kinases, activated at reperfusion (ERK1/2, Akt, PKC-ε and PKG).
  - leads to cytoprotection through induction of anti-apoptotic genes (e.g. *Bcl-2*), inhibition of pro-apoptotic genes (*BAX*/*BAD*), increased calcium uptake, activation of eNOS, activation of glycogen synthase kinase-3β (GSK-3β) and activation of the mitochondrial potassium ATP (K_ATP_) channel.
➢ SAFE pathway:
  - involves activation of the JAK-STAT cascade.
➢ RISK and SAFE pathways converge and inhibit the opening of the mitochondrial permeability transition pore (MPTP), potentially a final common effector pathway of tissue protection.
➢ iPOST also inhibits MPTP opening by delaying the normalization of intracellular pH.

**Box 2 Fig.**
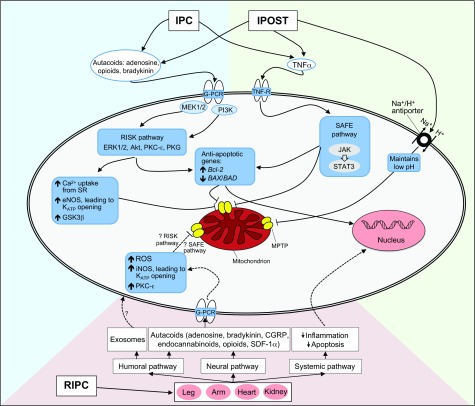
Source data derived from: Bromage et al., 2014; Giricz et al., 2014; Hausenloy, 2013; Lecour, 2009; Sluijter et al., 2014. Abbreviations: Akt, serine/threonine protein kinase, also known as protein kinase B (PKB); Bcl-2, B-cell lymphoma 2 gene; BAD, Bcl-2-associated death promoter; BAX, BCL2-associated X protein; eNOS, endothelial nitric oxide synthase; ERK 1/2, extracellular signal regulated kinases 1/2; G-PCR, G-protein coupled receptor; GSK3β, glycogen synthase kinase-3β; iNOS, inducible nitric oxide synthase; JAK, Janus kinase; K_ATP_, mitochondrial potassium ATP channel; MEK1/2, mitogen-activated protein kinase kinase; MPTP, mitochondrial permeability transition pore; PI3K, phosphatidylinositol-(4,5)-bisphosphate 3-kinase; PKCε, protein kinase Cε; PKG, cGMP-dependent protein kinase; ROS, reactive oxygen species; SDF-1α; stromal-cell-derived factor 1α; STAT3, signal transducer and activator of transcription 3; SR, sarcoplasmic reticulum; TNFα, tumour necrosis factor α; TNF-R, TNF receptor.

**RIPC mechanism**

➢ Signal thought to travel from the remote organ to tissues that are at risk via one of three proposed pathways: the humoral, the neural and/or the systemic pathway (Hausenloy and Yellon, 2008).
➢ Humoral pathway: factors are released in response to an RIPC stimulus and travel in the bloodstream to the organ at risk. Such factors include:
  - autacoids, including adenosine, opioids, endocannabinoids, bradykinin, CGRP and stromal-cell-derived factor 1α (SDF-1α) (Bromage et al., 2014);
  - extracellular vesicles (exosomes) (Giricz et al., 2014).
➢ Neural pathway: the RIPC protocol stimulates efferent nerves, which:
  - directly terminate on the organ at risk to trigger tissue protection;
  - release factors into the circulation to trigger tissue protection (see ‘Humoral pathway’).
➢ Systemic pathway:
  - RIPC signal generates a systemic anti-inflammatory/anti-apoptotic genomic response via upregulation of anti-inflammatory genes and inhibition of leukocyte activation (Saxena et al., 2010).
➢ The humoral, neural and systemic pathways are thought to act via intracellular signal transduction cascades. These are less well understood than those in IPC or iPOST but are thought to involve similar mediators, including nitric oxide (NO) and ROS signalling, and activation of PKC-ε. These are represented by dotted lines in the figure to indicate that the pathways are unclear at present.

Tsang et al. reported that, in the Goto-Kakizaki rat model of diabetes (a non-obese Wistar rat substrain that develops early type 2 diabetes), hearts could be preconditioned, but had a higher threshold for preconditioning. The diabetic animals required three cycles of IPC to achieve significant tissue protection following ischemic insult, whereas the non-diabetic controls required only one cycle (Tsang et al., 2005).

The reduced efficacy of IPC in the diabetic heart might be due to alterations to the components of the IPC signal transduction pathway; for example, reduced Akt phosphorylation (Tsang et al., 2005), mitochondrial K_ATP_ channel dysfunction (del Valle et al., 2002), activation of GSK-3β (see Box 1) (Yadav et al., 2010) and reduced NO availability (Gu et al., 2008).

### Studies in humans

In humans, diabetes is reported to block the preconditioning effects of pre-infarct angina in individuals with an acute myocardial infarction (Ishihara et al., 2001) and abrogate the effectiveness of IPC during coronary angioplasty (see Box 1); this disruption is proposed to be due to reduced activation of K_ATP_ channels, based on studies using glibenclamide (K_ATP_ channel antagonist) and nicorandil (activates K_ATP_ channels) (Lee and Chou, 2003). More recently, in an *ex vivo* human model, diabetes was shown to increase the threshold for preconditioning, owing to a reduction in Akt phosphorylation in the diabetic myocardium (Sivaraman et al., 2010).

Very few studies have examined the effects of diabetes on the ability of a tissue to respond to RIPC. In humans, following an RIPC stimulus (inflation of a blood-pressure cuff on the upper arm to 200 mmHg, 4×5-minute cycles), an undefined humoral factor is released by non-diabetic healthy individuals, but not by diabetic patients with neuropathy; this factor, when injected into rabbits, led to an increased tolerance to myocardial ischemia (Jensen et al., 2012). Similar findings were published using *ex vivo* human atrial tissue, which was only protected by a humoral factor from control and not from diabetic patients (Jensen et al., 2013).

As with IPC, most, but not all (Lacerda et al., 2012), studies in diabetic animals seem to confirm that diabetes represents a barrier to iPOST. Mice treated with streptozotocin (see Box 1) are unable to respond to an iPOST protocol due to a loss of iPOST-mediated ERK phosphorylation. However, the beneficial effects of iPOST are regained if blood sugars are normalized with islet-cell transplantation 2 weeks before the cardiac ischemia (Przyklenk et al., 2008b). Further studies have demonstrated abnormalities in other members of the signal pathways that abolish the effectiveness of iPOST in diabetic animals, including alterations to Jak-STAT signalling in Otsuka-Long-Evans-Tokushima fatty rats (Hotta et al., 2010), alterations to PI3K-Akt signalling (Liu et al., 2013), and reduced ERK1/2 phosphorylation and alteration in GSK-3β signalling in streptozotocin-treated rats (Gross et al., 2007) (see glossary of terms in Box 1).

In humans, indirect evidence for the failure of iPOST in diabetes comes from a large retrospective study of patients who underwent a PCI for an acute myocardial infarction, in which multiple balloon inflations acted as a post-conditioning stimulus. On subgroup analysis, the authors found that diabetes seemed to abolish the effects of the post-conditioning protocol (Yetgin et al., 2014).

## Hypertension and left ventricular hypertrophy

### The condition

Hypertensive heart disease is common and associated with the development of cardiac hypertrophy (Drazner, 2011). It is well established that the hypertrophied heart is more susceptible to ischemia (Anderson et al., 1987) and post-ischemic arrhythmias (McLenachan et al., 1987), and it is logical to assume that it might also alter the response to ischemic conditioning. In 2013, it was estimated that nearly a third of the United States population (78 million people) had high blood pressure (Go et al., 2013a). Hypertension is the most common comorbidity in patients presenting with an acute myocardial infarction (Wagner et al., 2013); 69% of patients with a first myocardial infarction and 77% with a first stroke are hypertensive (Go et al., 2013b).

### Effects on preconditioning strategies

Many animal studies have attempted to investigate whether the hypertrophied heart responds to ischemic conditioning, with differences being reported in the models, methods and results. Some authors initially reported that hypertensive rat hearts could be preconditioned: Boutros and Wang demonstrated a preserved response to IPC in an isolated heart model from spontaneously hypertensive rats (SHRs) (Boutros and Wang, 1995), whereas others demonstrated in an *in vivo* model of myocardial infarction that rats who developed left ventricular hypertrophy (LVH) in response to saline and a deoxycorticosterone-acetate hypertensive diet still benefited from IPC (Speechly-Dick et al., 1994). However, a subsequent study reported that hypertension abolished the cardioprotective effects of IPC in genetically hypertensive rats (Moolman et al., 1997).

Little is known of the effect of RIPC on the hypertrophied heart. A study using fludrocortisone and a high-salt diet to induce cardiac hypertrophy in an *ex vivo* rat model of myocardial ischemia surprisingly suggested that RIPC could protect hypertrophied rodent hearts, but not control hearts (Voucharas et al., 2011).

As with IPC, the effects of iPOST in the hypertrophied myocardium demonstrate conflicting results, depending on the model of LVH used. Isolated SHR hearts display a reduced response to the cardioprotective effects of iPOST (Penna et al., 2010), with a loss of GSK-3β phosphorylation as the likely mechanism (Wagner et al., 2013). Isolated, hypertrophied rat hearts, produced by chronic nandrolone treatment, were also found to be resistant to iPOST (Penna et al., 2010). Although the investigators did not find any difference in GSK-3β phosphorylation, they did note reduced Akt phosphorylation in the hypertrophied group. Conversely, when chronic treatment with angiotensin II was used to develop a model of LVH in rats (Hernández-Reséndiz et al., 2013), the beneficial effects of iPOST were preserved. One explanation for the differing results observed in these different disease models is that SHRs are also known to have dyslipidemia and impaired glucose tolerance, making their failure to respond to iPOST difficult to attribute entirely to hypertension (van den Brandt et al., 2000).

### Studies in humans

Further evidence that the protection conferred by IPC is lost in the context of hypertension comes from clinical research in which the IPC effect of prodromal angina (preceding the actual infarct event) on subsequent infarct size reduction was lost in patients with hypertension (Takeuchi et al., 2011). Similarly, in another study of patients presenting with an NSTEMI (see Box 1 for a glossary of terms) with pre-infarct angina, the protective effects of IPC were lost in a subgroup of hypertensive patients, although whether these patients also had LVH was not stated (Lorgis et al., 2012).

## Obesity

### The condition

Obesity is increasingly a global epidemic affecting over 1 billion people worldwide, with numbers forecast to increase over the next 20 to 30 years (Ghoorah et al., 2014). As adipose tissue accumulates, there are structural and functional changes in the myocardium that predispose individuals to adverse outcomes, and obesity has been shown to be an independent risk factor for cardiovascular disease, heart failure and sudden death (Poirier et al., 2006).

### Effects on preconditioning strategies

Many investigations into preconditioning in obesity have been performed in the context of type 2 diabetes and the metabolic syndrome. Aside from insulin resistance, it would seem, however, that IPC is negatively affected by obesity. Evidence for this comes from work that reported that, although both obese and lean insulin-resistant rats were resistant to IPC, post-ischemic recovery was impaired in obese animals compared with lean animals (Kristiansen et al., 2004). In addition, in the context of myocardial injury, when compared with lean Zucker rats, normoglycemic obese Zucker rats were unable to respond to either ischemic or chemical preconditioning, due to increased mitochondrial oxidative stress and the impaired activation of mitochondrial K_ATP_ (Katakam et al., 2007). Conversely, obesity was not a barrier to RIPC in a mouse model of ischemic liver injury (O’Leary et al., 2011).

Lack of cardioprotection by iPOST has also been demonstrated in *ob/ob* leptin-deficient mice, through a reduction in phosphorylation in members of the RISK pathway (see Box 1) (Bouhidel et al., 2008). However, the absence of leptin, which is known to have a direct cardioprotective effect, might confound these results (Smith et al., 2006). Similarly, in rats with the metabolic syndrome, a lack of GSK-3β and ERK phosphorylation have been linked to their inability to respond to iPOST (Wagner et al., 2008).

### Studies in humans

To date, there have been no clinical studies published that explore the effects of obesity on ischemic conditioning in humans.

## Senescence

### The condition

Age confers significant structural and functional changes in the myocardium, including reduced ischemia tolerance (Juhaszova et al., 2005), and has a significant impact on prognosis following an acute myocardial infarction (Tofler et al., 1988).

### Effects on preconditioning strategies

The effects of preconditioning on the aged heart were investigated early in the quest to better understand this cardioprotective phenomenon. In 1996, isolated rat hearts were used to demonstrate that the effects of IPC were lost in the ageing heart, through a reduction in norepinephrine and α-adrenergic receptor activation in response to IPC (Abete et al., 1996), or due to the reduced translocation of protein kinase C (see Box 2) (Tani et al., 2001). These results have been substantiated in similar studies in rats, which have shown that the beneficial effects of IPC on the morphological consequences of ischemia were also lost in older rats (Ebrahim et al., 2007; Fenton et al., 2000; Schulman et al., 2001). Furthermore, as with diabetes, the effects of ageing in ‘middle aged’ rats could be overcome by additional cycles of IPC (Schulman et al., 2001).

Conversely, a study of isolated hearts from older rabbits reported no loss in the efficacy of IPC (Przyklenk et al., 2001), although there was criticism that the animals used were not sufficiently aged to represent a true model of senescence (Abete et al., 2001). Equally, a similar study of senescent sheep found that IPC-induced myocardial infarct size reduction was well preserved despite age (Burns et al., 1996).

There is little published data about RIPC in animal models of senescence. One study looking at the effect of both local and remote IPC on endothelial protection and reactivity in older human patients found that the beneficial effects seemed to be preserved, as measured by flow-mediated dilatation (Moro et al., 2011).

Ageing does seem to reduce the beneficial effects of iPOST. In senescent mice, the protective effects of iPOST on myocardial ischemia are attenuated, with a proposed explanation being a decrease in ERK phosphorylation and an increase in MKP-1 (mitogen-activated protein kinase phosphatase-1; see Box 1) expression in the aged heart (Przyklenk et al., 2008a). Furthermore, the response to iPOST in the ageing mouse heart seems to depend on the protocol of iPOST used, and a deficiency in STAT-3 associated with ageing might contribute to the dampened response (Boengler et al., 2008).

### Studies in humans

In humans, it was reported in a retrospective study that the protective effect of pre-infarct angina was lessened in elderly patients (Abete et al., 1997). *Ex vivo* studies of human atrial tissue demonstrate conflicting results: one group published a resistance to IPC with senescence (Bartling et al., 2003), whereas others reported no resistance (Loubani et al., 2003).

No human studies have specifically examined the effect of age on the ability to post-condition. However, most patients enrolled in clinical trials of iPOST in cardiovascular disease are over 50 years of age, suggesting that at least middle-aged humans can benefit from iPOST.

## Chronic kidney disease

### The condition

An underlying chronic kidney disease (CKD; see Box 1) is associated with reduced myocardial ischemia tolerance (Byrne et al., 2012; Dikow et al., 2004) and is a common comorbidity in individuals with heart disease: over 25% of patients presenting with an acute coronary syndrome have stage 3 CKD or worse (Medi et al., 2009). Patients with CKD have a two to four times increased cardiovascular risk, even adjusting for traditional risk factors (Gansevoort et al., 2013), and impaired renal function in patients with acute myocardial infarction is an important and consistent predictor of mortality, at least up to 10 years following the event (Smith et al., 2008).

### Effects on preconditioning strategies

Surprisingly, CKD does not seem to be a barrier to ischemic conditioning. We have reported that, in the subtotal nephrectomy rodent model of moderate CKD in which we induced myocardial ischemia, underlying CKD led to an increased myocardial infarct size but did not alter the tissue protective effects of IPC, RIPC and iPOST (Byrne et al., 2012). In addition, even when underlying uremia was prolonged for over 6 months, the ability of the myocardium to respond to an IPC stimulus remained intact (Kocsis et al., 2012).

### Studies in humans

Human trial data have confirmed that patients with moderate CKD can respond to an RIPC stimulus (Er et al., 2012). Additional clinical trials (The Context trial, clinicaltrials.gov NCT01395719, and the REPAIR Trial, ISRCTN30083294) are underway in the field of renal transplantation, which will hopefully provide definitive data on the ability to precondition tissues in the context of CKD.

## Gender

### Effects on preconditioning strategies

The ‘default’ gender used in animal studies of myocardial ischemia is male. Although gender does not represent a comorbidity as such, it does have significant effects on both tolerance to ischemia and response to ischemic conditioning strategies. Oestrogen has several beneficial effects in the context of myocardial ischemia tolerance, which might confound findings: it activates mitochondrial K_ATP_ channels in a canine model of myocardial ischemia (Lee et al., 2000); reduces calcium overload during ischemia in an *in vitro* mouse model of simulated cardiomyocyte ischemia (Sugishita et al., 2001); increases NO production in dogs (Node et al., 1997); reduces apoptosis in rat cardiomyocytes (Pelzer et al., 2000); reduces free radical generation in a canine model of myocardial ischemia (Kim et al., 1996); and reduces post-infarction inflammation in a rat model of myocardial ischemia (Squadrito et al., 1997). These effects could partly explain the lower incidence of cardiac events in premenopausal women compared with men (Khalil, 2013).

The impact of gender on ischemic conditioning is complex, often contradictory, and seems to depend on the organ studied and the duration of the insult. In a study in mice, female hearts were more resistant to ischemia and reperfusion injury than male hearts, but were unable to respond to an IPC protocol (Song et al., 2003). However, the effects of gender on IPC do not seem to totally depend on sex hormones, because female rats remained resistant to IPC even after gonadectomy. In contrast, others have reported that female rats responded to an IPC stimulus, but this response was blocked following gonadectomy and then re-established with chronic oestrogen administration (Shinmura et al., 2008). Such differences might also be organ-specific, with data reporting that gender did not have an impact on the ability of rat kidneys to respond to IPC (Ebrahimi et al., 2012).

Again, there is very little data specifically regarding the impact of gender in the context of RIPC. The effects of gender on myocardial ischemia tolerance and iPOST in rats seem to be complicated. A study by Penna et al. involving an *ex vivo* rat model of myocardial ischemia, in which a short ischemia of 10 minutes duration was followed by 2 hours of reperfusion, showed that such treatment led to larger infarct sizes in female hearts than in male hearts (Penna et al., 2009). However, this effect was reversed when the ischemia duration was lengthened to 30 minutes, with females having less injury than males (Penna et al., 2009). The gender-dependent effects of iPOST also seem to be time-dependent, with only female rat hearts responding to iPOST following a brief exposure to ischemia, whereas both genders were protected by iPOST following ischemia lasting 30 minutes, but male rats to a greater extent (Crisostomo et al., 2006; Penna et al., 2009). Similar findings were reported in rabbits, where iPOST seems to be less effective in females after prolonged ischemia, although protection is reinstated following ovariectomy (Demerouti et al., 2013).

### Studies in humans

There is conflicting evidence that gender plays a role in human post-conditioning. Some studies report that men respond better to iPOST than do women (Zhou et al., 2012), whereas others report the converse (Yetgin et al., 2014).

## Medication use

Several common medications, including statins (Morales-Villegas et al., 2011), nicorandil (Sakai et al., 2002), sildenafil (Kukreja et al., 2005), erythropoietin (Baker, 2005), opiates (Murphy et al., 2006) and cyclosporine (Piot et al., 2008), have been shown to mimic the effects of ischemic conditioning, leading to tissue protection through ‘pharmacological conditioning’. For details of all medications reported in this section and their clinical use, please refer to Box 3.

**Box 3. Glossary of medications**

Brief summary of each drug mentioned in the ‘Medication use’ section: the type of drug, its mechanism of action and its clinical use.

Aminophylline: non-selective phosphodiesterase inhibitor and adenosine receptor antagonist used as a bronchodilator in the treatment of asthma. (**Bamiphylline** is a selective A1 adenosine receptor antagonist.)
Captopril: is an angiotensin-converting enzyme (ACE) inhibitor used in the treatment of heart failure and hypertension. (**Enalaprilat** is the active enzyme of enalapril, another ACE inhibitor.)
Carvedilol: blocks both β- and α-adrenergic activity and is used in the treatment of hypertension and congestive cardiac failure.
Cyclosporine: a calcineurin inhibitor that blocks the actions of T cells; used widely as an immunosuppressant, particularly in organ transplantation (e.g. kidney, heart, bone marrow).
Erythropoietin: endogenously produced by the kidneys to stimulate red blood cell production, this is given in a recombinant form to patients with chronic kidney disease (in which endogenous production is reduced) or other conditions that cause anemia.
Naloxone: a pure opioid antagonist and is used to reverse overdoses of opiates.
Opiates: psychoactive and analgesic drugs that work by binding opioid receptors in the nervous system; examples include morphine and fentanyl.
Phentolamine: a vasodilator working through α-adrenergic blockade; used in hypertensive emergencies.
Pioglitazone: a thiazolidinedione PPARγ agonist that modulates the transcription of insulin-sensitive genes and is used in the treatment of type 2 diabetes.
Sildenafil: vasoactive drug by means of increasing cGMP; used in erectile dysfunction and pulmonary artery hypertension.
Statins: HMG co-reductase inhibitors, widely used to lower cholesterol both as primary and secondary prevention of cardiovascular events. Examples include **atorvastatin**, simvastatin and **pravastatin**.
Sulfonylureas: stimulate the pancreas to make more insulin and are used in the treatment of type 2 diabetes. Examples include gliclazide, **glibenclamide** and **glimepiride**.

The long-term use of oral sulfonylureas has been shown, in human studies, to block the cardioprotective effect of IPC through the inhibition of K_ATP_ channels (Cleveland et al., 1997). Aminophylline and bamiphylline have also been found to inhibit IPC in human studies, suggesting, therefore, that adenosine receptors are involved in the cardioprotective effect (Carr et al., 1997; Claeys et al., 1996; Tomai et al., 1996).

The abrogating effects of naloxone on IPC (Tomai et al., 1999), as well as of phentolamine (Tomai et al., 1997), have also been studied in humans, suggesting roles for opioid and α-adrenergic receptors, respectively, in the mechanisms of IPC protection. In addition, several common medications have been shown to restore the effects of IPC, which are lost due to an underlying comorbidity. For example, pravastatin was able to restore the effectiveness of IPC lost in hypercholesterolemic rabbits (Ueda et al., 1999). Similarly, pioglitazone restored the anti-arrhythmic effects of IPC lost in diabetic rats (Sasaki et al., 2007). Furthermore, glimepiride restored the cardioprotective effects of IPC in diabetic rats, independent of blood glucose (Hausenloy et al., 2013). Carvedilol has been shown to restore the effects of IPC in a rat model of coronary artery stenosis (Oikawa et al., 2008), and humans resistant to IPC were rendered responsive to IPC by pre-treatment with enalaprilat (Ungi et al., 2008). Mensah et al. suggested that atorvastatin, a commonly used medication in patients with cardiovascular disease, had an acute cardioprotective effect in rats through activation of PI3K-Akt-eNOS signalling, but that this was lost with chronic use via upregulation of PTEN (see Box 1). However, cardioprotection could be restored with high-dose atorvastatin in addition to chronic use in the immediate pre-ischemic setting, which offers a new potential clinical avenue (Mensah et al., 2005).

There is little published data on the effects of medication use in the context of RIPC. However, the protective benefits of iPOST were reported to be lost in rats already chronically treated with captopril. However, captopril had already conferred a beneficial effect to those animals through reduced ventricular hypertrophy and reduced susceptibility to IRI (Penna et al., 2010). In addition, underlying coronary artery stenosis abolished the effects of iPOST in rats, which could be rescued by treatment with carvedilol (Oikawa et al., 2008).

## Other factors affecting the efficacy of ischemic conditioning strategies

**Fig. 2.**
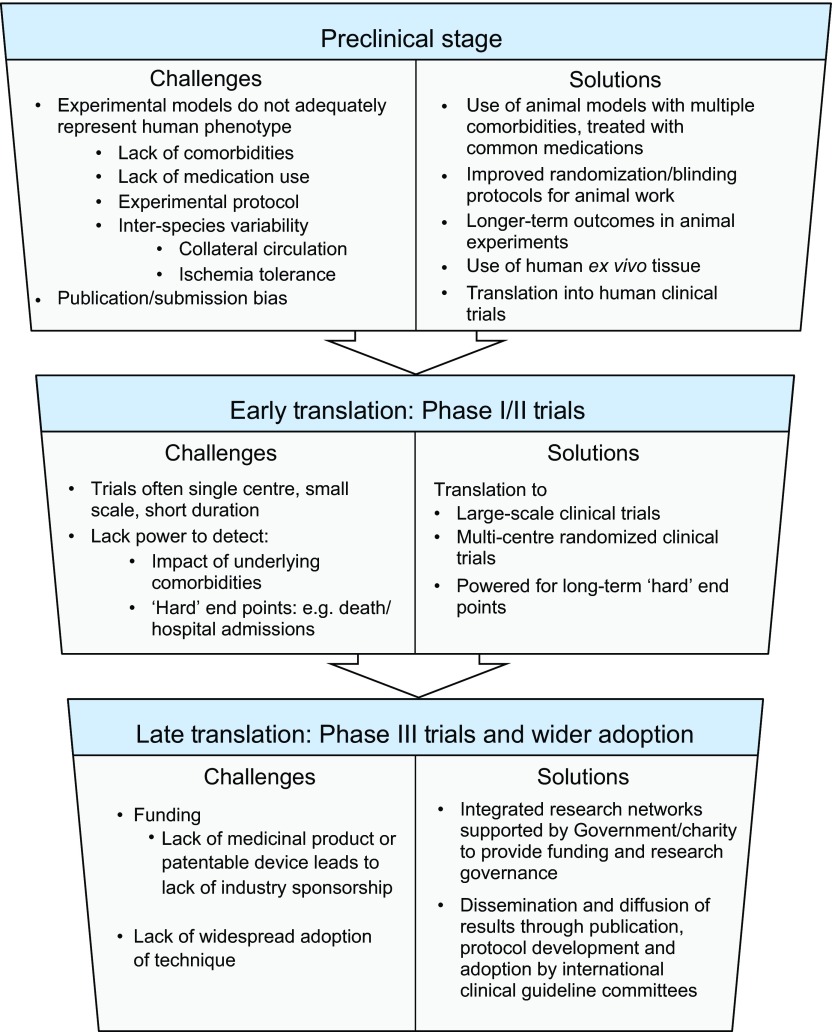
**Challenges and potential solutions to translating ischemic conditioning studies from ‘bench to bedside’.** Flowchart summarizing the main challenges related to the testing of ischemic conditioning strategies in animal studies (preclinical stage) and to the translation of these approaches into clinical trials (early translation stage, Phase I/II trials). Possible solutions to optimize both the preclinical research stage and the planning of clinical trials are also shown. Should a particular strategy of ischemic conditioning successfully pass the Phase I/II clinical trial phases, future challenges to wider diffusion (late translation stage) include the need for guideline development and adoption of standardized protocols by national and international guideline bodies.

It is clear that a number of common comorbidities impact the effectiveness of ischemic conditioning strategies and should be taken into consideration when designing future translational studies. However, there might also be other fundamental explanations for the disparity seen between animal studies and clinical trials (see Fig. 2). One key difference is the use of non-comorbid, genetically homogenous, juvenile animals, of the same sex, housed together and fed identical diets. In animal models of ischemia-reperfusion, the timing of ischemia is precisely known, unlike much of the work in humans, in whom the onset of tissue ischemia is either unknown or imprecisely known. Although the exact duration of ischemia is of crucial importance to tissue injury, this duration varies widely depending on the animal model used: with smaller animals, such as rats, 30 minutes of myocardial ischemia is sufficient to lead to infarct sizes of around 50% of the at-risk area, whereas, in larger animals, such as pigs or primates, up to 90 minutes of ischemia is required to lead to similar degrees of tissue injury (Ludman et al., 2010). There are also differences in how the myocardium is rendered ischemic between animal and human studies. In animals, ischemia is induced by external occlusion of an otherwise normal artery, usually by the same skilled operator in each case. In humans, an unstable plaque ruptures, leading to a variable degree of staggered occlusion caused by clot formation over the ulcerated plaque, and showering debris into the bloodstream, which leads to an inflammatory response and distal embolization.

Furthermore, coronary collateral circulation, whereby some myocardial tissue is perfused by more than one arterial tree, might alter an individual’s response to ischemia. Watershed areas between the zones of two arterial territories can rely on flow from nearby vascular territories during ischemia. Collateral circulation is profoundly species-dependent, with pigs, rats and primates having a low collateral circulation, whereas dogs and guinea pigs have significant collateralization (Verdouw et al., 1998). In humans, up to one third of individuals who present with an ischemic event have evidence of collateral coronary flow (Seiler, 2010), which is associated with smaller infarct size and with better long-term outcomes (Meier et al., 2013). The volume of tissue rendered ischemic is also an important determinant of infarct size (Christian et al., 1992); this is easily quantifiable in animal studies by using dye techniques (Byrne et al., 2012) but is much more difficult to accurately establish in humans (Bøtker et al., 2012). In addition, the validity of animal models as a tool to study genomic and transcriptomic responses following injury has been cast in doubt after recent data show that the murine genomic response to shock is fundamentally different to that of humans (Seok et al., 2013).

The isolated perfused animal heart (Langendorff) model has been used for decades to study the effects of myocardial IRI. However, this *ex vivo* model uses buffered crystalloid solutions rather than blood to perfuse the heart and does not involve an intact nervous system. This is clearly important when studying the effects of RIPC; hormonal and neural pathways are involved in the RIPC response and, in a model that does not incorporate access to the cirulation or neuronal signalling, it is difficult to interpret or extrapolate results to a human clinical level. The open-chest model of myocardial ischemia (Wayman et al., 2003) addresses these issues but is more technically challenging to perform, with not infrequent animal death from myocardial arrhythmias and hypotension (Dikow et al., 2004). This could lead to selection bias, with only animals that are healthier or less likely to have an arrhythmic or hypotensive episode being included.

Finally, a publication and/or submission bias might lead to relatively fewer animal studies with negative results being published compared with the publication of clinical trials reporting negative results. This publication bias in animal models has been quantified in the neurological literature (Tsilidis et al., 2013). Further bias might arise in animal studies from less rigorous blinding controls and randomization compared with human clinical trials, which are conducted under explicit research governance structures.

## Conclusion and future perspectives

The abrogating effects on ischemic conditioning of the various comorbidities covered in this Review are not inconsiderable. The reality is that many of the patients who might benefit most from cardioprotective strategies will also have other significant disease burdens that cannot be ignored. To plan future successful clinical trials, this must be taken into account. Further studies are needed to clearly define optimal preconditioning strategies in animal models with these comorbidities. It is not sufficient only to conclude that these various comorbidities diminish the effects of preconditioning: that this is the case is now well established. Rather, a more focused approach is now required to look at clear strategies to overcome this phenomenon. This must involve focusing on defining the optimal protocol for IPC, RIPC or iPOST in comorbid animal models, or looking at concomitant treatment with drugs that have already been shown to help increase the response to these interventions. Furthermore, our review highlights the relative lack of experimental data looking at RIPC in comorbid animal models, which is arguably the most attainable strategy to take forward into human studies.

It is not currently clear what the ideal human conditioning protocol is, in terms of duration, number of conditioning cycles and, importantly, whether this optimal protocol varies depending on which conditioning strategy is used. Future clinical trials taking these comorbidities into consideration should be of an adequate size and duration to provide statistical power to look at important clinical end points, and potentially change routine clinical practice (Fig. 2). Two very large clinical trials are currently underway: the ERICCA trial (Effect of Remote Ischemic Preconditioning on Clinical Outcomes in Patients Undergoing Coronary Artery Bypass Graft Surgery; ClinicalTrials.Gov NCT01247545), aiming to recruit over 1500 patients, and the RIP-Heart study (Remote Ischemic Preconditioning for Heart Surgery; ClinicalTrials.Gov NCT01067703), aiming to recruit over 2000 patients, which will hopefully answer many of these important questions.

It is tempting to see the impact of comorbidities on ischemic conditioning as a failure of translation. However, with a greater understanding of the limitations of animal models and of how comorbidities can modulate the effectiveness of ischemic conditioning strategies comes an opportunity to understand better how these undoubtedly safe, cheap and potentially crucially important interventions confer tissue protection. With our greater appreciation of the strengths and limitations of the differing ischemic conditioning strategies, we will be able to better design randomized clinical trials using ischemic conditioning strategies to improve outcomes for those patients who are likely to benefit most.
